# Supplementary material for: A Systematic Review of Dietary Interventions for Cancer Survivors and Their Families or Caregivers
Source: Nutrients. 2023 Dec 23;16(1):56. doi: 10.3390/nu16010056 (PMC10780967; doi:10.3390/nu16010056)
Supplement: Supplementary file 1 [file nutrients-16-00056-s001.zip › nutrients-2777692-supplementary.pdf]

Supplementary Table S1. Search Terms

PubMed	Scopus	CINAHL
(("Family"[Mesh] OR "Caregivers"[Mesh] OR family[tiab] OR families[tiab] OR caregiver[tiab] OR caregivers[tiab] OR carer[tiab] OR carers[tiab] OR "care giver"[tiab] OR "care givers"[tiab] OR caregiving[tiab] OR "care giving"[tiab] OR relatives[tiab] OR parent[tiab] OR parents[tiab] OR mother[tiab] OR mothers[tiab] OR father[tiab] OR fathers[tiab] OR siblings[tiab] OR sibling[tiab] OR sisters[tiab] OR sister[tiab] OR brothers[tiab] OR brother[tiab] OR grandparent[tiab] OR grandparents[tiab] OR Dyad*[tiab] OR triad*[tiab] OR spouses[tiab] OR spouse[tiab] OR partners[tiab] OR partner[tiab] OR husband[tiab] OR husbands[tiab] OR wife[tiab] OR wives[tiab] OR surrogate[tiab] OR surrogates[tiab] OR stepfamily[tiab] OR stepfamilies[tiab] OR "patient advocate"[tiab] OR "patient advocates"[tiab] OR proxy[tiab] OR "significant other"[tiab] OR "significant others"[tiab]) AND ("Diet"[Mesh] OR "Nutrition Therapy"[Mesh] OR "Nutrition Assessment"[Mesh] OR "Overnutrition"[Mesh:NoExp] OR "Malnutrition"[Mesh] OR "Feeding Behavior"[Mesh] OR "Food"[Mesh] OR nutrition[tiab] OR nutritional[tiab] OR nutrient[tiab] OR nutrients[tiab] OR micronutrient[tiab] OR micronutrients[tiab] OR calorie[tiab] OR calories[tiab] OR caloric[tiab] OR food[tiab] OR foods[tiab] OR feeding[tiab] OR eating[tiab] OR diet[tiab] OR diets[tiab] OR dietary[tiab] OR dieting[tiab] OR overnutrition[tiab] OR hypernutrition[tiab] OR malnutrition[tiab] OR malnourishment[tiab] OR undernutrition[tiab] OR fats[tiab] OR carbohydrates[tiab] OR protein[tiab] OR proteins[tiab] OR fruit[tiab] OR fruits[tiab] OR	TITLE-ABS-KEY((family OR families OR caregiver OR caregivers OR carer OR carers OR "care giver" OR "care givers" OR caregiving OR "care giving" OR relatives OR parent OR parents OR mother OR mothers OR father OR fathers OR siblings OR sibling OR sisters OR sister OR brothers OR brother OR grandparent OR grandparents OR Dyad* OR triad* OR spouses OR spouse OR partners OR partner OR husband OR husbands OR wife OR wives OR surrogate OR surrogates OR stepfamily OR stepfamilies OR "patient advocate" OR "patient advocates" OR proxy OR "significant other" OR "significant others") AND (nutrition OR nutritional OR nutrient OR nutrients OR micronutrient OR micronutrients OR calorie OR calories OR caloric OR food OR foods OR feeding OR eating OR diet OR diets OR dietary OR dieting OR overnutrition OR hypernutrition OR malnutrition OR malnourishment OR undernutrition OR fats OR carbohydrates OR protein OR proteins OR fruit OR fruits OR vegetable OR vegetables OR fiber OR fibre OR dairy OR meat OR meats OR salt OR sugar) AND (Neoplasms OR Neoplasm OR Neoplasia OR Cancer OR Cancers OR cancerous OR Tumor OR Tumors OR Tumour OR Tumours	(MH "Family+" OR MH "Caregivers" OR TI (family OR families OR caregiver OR caregivers OR carer OR carers OR "care giver" OR "care givers" OR caregiving OR "care giving" OR relatives OR parent OR parents OR mother OR mothers OR father OR fathers OR siblings OR sibling OR sisters OR sister OR brothers OR brother OR grandparent OR grandparents OR Dyad* OR triad* OR spouses OR spouse OR partners OR partner OR husband OR husbands OR wife OR wives OR surrogate OR surrogates OR stepfamily OR stepfamilies OR "patient advocate" OR "patient advocates" OR proxy OR "significant other" OR "significant others") OR AB (family OR families OR caregiver OR caregivers OR carer OR carers OR "care giver" OR "care givers" OR caregiving OR "care giving" OR relatives OR parent OR parents OR mother OR mothers OR father OR fathers OR siblings OR sibling OR sisters OR sister OR brothers OR brother OR grandparent OR grandparents OR Dyad* OR triad* OR spouses OR spouse OR partners OR partner OR husband OR husbands OR wife OR wives OR surrogate OR surrogates OR stepfamily OR stepfamilies OR "patient advocate" OR "patient advocates" OR proxy OR "significant other" OR "significant others")) AND (MH "Diet+" OR MH "Diet Therapy+" OR MH "Nutritional Assessment" OR MH "Overnutrition" OR MH "Malnutrition" OR MH "Eating Behavior+" OR MH "Food+" OR TI (nutrition OR nutritional OR nutrient OR nutrients OR micronutrient OR micronutrients OR calorie OR calories OR caloric OR food OR foods OR feeding OR eating OR diet OR diets OR dietary OR dieting OR overnutrition OR hypernutrition OR malnutrition OR malnourishment OR undernutrition OR fats OR carbohydrates OR protein OR proteins OR fruit OR fruits OR vegetable OR vegetables OR fiber OR fibre OR dairy OR meat OR meats OR salt OR sugar) OR AB (nutrition OR nutritional OR nutrient OR nutrients OR micronutrient OR micronutrients OR calorie OR calories OR caloric OR food OR foods OR feeding OR eating OR diet OR diets OR dietary OR dieting OR overnutrition OR hypernutrition OR malnutrition OR malnourishment OR

vegetable[tiab] OR vegetables[tiab] OR fiber[tiab] OR fibre[tiab] OR dairy[tiab] OR meat[tiab] OR meats[tiab] OR salt[tiab] OR sugar[tiab])) AND (Neoplasms[Mesh] OR Neoplasms[tiab] OR Neoplasm[tiab] OR Neoplasia[tiab] OR Cancer[tiab] OR Cancers[tiab] OR cancerous[tiab] OR Tumor[tiab] OR Tumors[tiab] OR Tumour[tiab] OR Tumours[tiab] OR carcinoma[tiab] OR carcinomas[tiab] OR malignancy[tiab] OR malignancies[tiab] OR malignant[tiab])) AND (survivors or survivor or survivorship))	OR carcinoma OR carcinomas OR malignancy OR malignancies OR malignant) AND (survivors or survivor or survivorship))	undernutrition OR fats OR carbohydrates OR protein OR proteins OR fruit OR fruits OR vegetable OR vegetables OR fiber OR fibre OR dairy OR meat OR meats OR salt OR sugar)) AND (MH "Neoplasms+" OR TI (Neoplasms OR Neoplasm OR Neoplasia OR Cancer OR Cancers OR cancerous OR Tumor OR Tumors OR Tumour OR Tumours OR carcinoma OR carcinomas OR malignancy OR malignancies OR malignant) OR AB (Neoplasms OR Neoplasm OR Neoplasia OR Cancer OR Cancers OR cancerous OR Tumor OR Tumors OR Tumour OR Tumours OR carcinoma OR carcinomas OR malignancy OR malignancies OR malignant)) AND (MH "Survivors+" OR TI (survivors or survivor or survivorship) OR AB (survivors or survivor or survivorship))

Supplementary Table S2. Quality assessment of the included reports excluding protocols (N=13).

Randomized Controlled Trials							
Checklist	Carmack (2021)	Crane (2021)	Denmark-Wahnefried (2014)	Denmark-Wahnefried (2023)	James (2015)	Manne (2021)	
1. Was the study described as randomized, a randomized trial, a randomized clinical trial, or an RCT?	Y	Y	Y	Y	Y	Y	
2. Was the method of randomization adequate (i.e., use of randomly generated assignment)?	N	Y	Y	Y	Y	Y	
3. Was the treatment allocation concealed (so that assignments could not be predicted)?	Y	Y	Y	Y	Y	Y	
4. Were study participants and providers blinded to treatment group assignment?	N	NR	N	N	N	N	
5. Were the people assessing the outcomes blinded to the participants' group assignments?	Y	Y	NR	NR	NR	NR	
6. Were the groups similar at baseline on important characteristics that could affect outcomes (e.g., demographics, risk factors, co-morbid conditions)?	Y	Y	Y	Y	Y	Y	

7. Was the overall drop-out rate from the study at endpoint 20% or lower of the number allocated to treatment?	Y	N	Y	Y	N	Y	
8. Was the differential drop-out rate (between treatment groups) at endpoint 15 percentage points or lower?	Y	N	Y	Y	Y	Y	
9. Was there high adherence to the intervention protocols for each treatment group?	Y	Y	N	Y	Y	Y	
10. Were other interventions avoided or similar in the groups (e.g., similar background treatments)?	Y	Y	Y	Y	Y	Y	
11. Were outcomes assessed using valid and reliable measures, implemented consistently across all study participants?	Y	Y	Y	Y	Y	Y	
12. Did the authors report that the sample size was sufficiently large to be able to detect a difference in the main outcome between groups with at least 80% power?	Y	N	Y	Y	Y	Y	
13. Were outcomes reported or subgroups analyzed prespecified (i.e., identified before analyses were conducted)?	Y	Y	Y	Y	Y	Y	
14. Were all randomized participants analyzed in the group to which they were originally assigned, i.e., did they use an intention-to-treat analysis?	Y	Y	Y	Y	Y	Y	
Total	12	10	11	12	11	12	
Checklist	Anton (2013)	Conlon (2016)	Dorfman (2022)	Knobf (2018)	Krouse (2017)	Stacey (2017)[†]	Stoutenberg (2016)
1. Was the study question or objective clearly stated?	Y	Y	Y	Y	Y	Y	Y
2. Were eligibility/selection criteria for the study population prespecified and clearly described?	Y	Y	Y	Y	Y	Y	Y
3. Were the participants in the study representative of those who would be eligible for the test/service/intervention in the general or clinical population of interest?	Y	Y	Y	Y	Y	Y	Y
4. Were all eligible participants that met the	N	NR	NR	N	N	N	NR

prespecified entry criteria enrolled?							
5. Was the sample size sufficiently large to provide confidence in the findings?	Y	N	N	N	N	NR	N
6. Was the test/service/intervention clearly described and delivered consistently across the study population?	Y	Y	Y	Y	Y	Y	Y
7. Were the outcome measures prespecified, clearly defined, valid, reliable, and assessed consistently across all study participants?	Y	Y	Y	Y	Y	Y	Y
8. Were the people assessing the outcomes blinded to the participants' exposures/interventions?	CD	N	N	N	N	NR	N
9. Was the loss to follow-up after baseline 20% or less? Were those lost to follow-up accounted for in the analysis?	Y	N	Y	N	N	Y	N
10. Did the statistical methods examine changes in outcome measures from before to after the intervention? Were statistical tests done that provided p values for the pre-to-post changes?	N	Y	Y	Y	Y	Y	Y
11. Were outcome measures of interest taken multiple times before the intervention and multiple times after the intervention (i.e., did they use an interrupted time-series design)?	N	N	N	N	N	N	N
12. If the intervention was conducted at a group level (e.g., a whole hospital, a community, etc.) did the statistical analysis take into account the use of individual-level data to determine effects at the group level?	NA	NA	NA	NA	NA	NA	NA
Total	7	6	7	6	6	7	6

Y=Yes, N=No, NR=not reported, CD=cannot determine, NA=Not applicable

† This study only analyzed the within-group pre-to-post changes from 20-week follow-up to 12-month follow-up among cancer survivors enrolled in the intervention group of James et al (2015) study. No between-arm comparisons. No intervention was conducted between two follow-up time points.
